# Structural and Functional Characterization of the ABCC6 Transporter in Hepatic Cells: Role on PXE, Cancer Therapy and Drug Resistance

**DOI:** 10.3390/ijms22062858

**Published:** 2021-03-11

**Authors:** Faustino Bisaccia, Prashant Koshal, Vittorio Abruzzese, Maria Antonietta Castiglione Morelli, Angela Ostuni

**Affiliations:** Department of Sciences, University of Basilicata, 85100 Potenza, Italy; faustino.bisaccia@unibas.it (F.B.); prashantkoshal240@gmail.com (P.K.); v.abruzz@hotmail.it (V.A.); maria.castiglione@unibas.it (M.A.C.M.)

**Keywords:** ABCC6, TNAP, *NT5E*, Pseudoxanthoma elasticum (PXE), cancer

## Abstract

Pseudoxanthoma elasticum (PXE) is a complex autosomal recessive disease caused by mutations of ABCC6 transporter and characterized by ectopic mineralization of soft connective tissues. Compared to the other ABC transporters, very few studies are available to explain the structural components and working of a full ABCC6 transporter, which may provide some idea about its physiological role in humans. Some studies suggest that mutations of *ABCC6* in the liver lead to a decrease in some circulating factor and indicate that PXE is a metabolic disease. It has been reported that ABCC6 mediates the efflux of ATP, which is hydrolyzed in PPi and AMP; in the extracellular milieu, PPi gives potent anti-mineralization effect, whereas AMP is hydrolyzed to Pi and adenosine which affects some cellular properties by modulating the purinergic pathway. Structural and functional studies have demonstrated that silencing or inhibition of ABCC6 with probenecid changed the expression of several genes and proteins such as *NT5E* and TNAP, as well as Lamin, and CDK1, which are involved in cell motility and cell cycle. Furthermore, a change in cytoskeleton rearrangement and decreased motility of HepG2 cells makes ABCC6 a potential target for anti-cancer therapy. Collectively, these findings suggested that ABCC6 transporter performs functions that modify both the external and internal compartments of the cells.

## 1. Introduction

**Figure 1:**
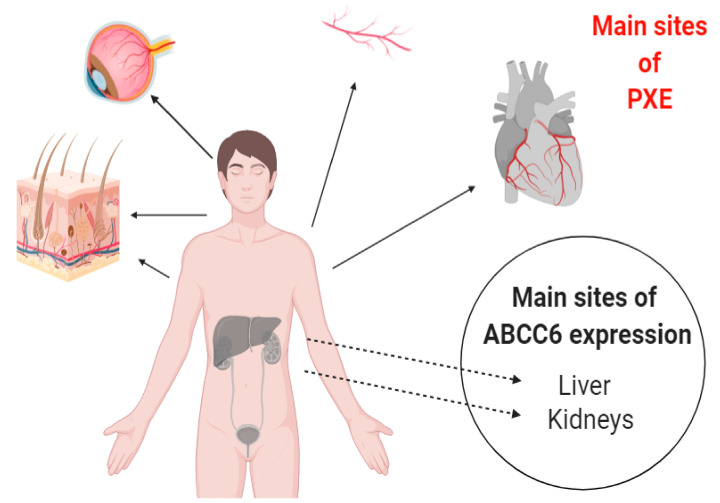
Pictorial presentation of ABCC6 expression and affected tissues in Pseudoxanthoma elasticum (PXE). The protein is expressed mainly in liver and kidney cells but the main areas involved in ectopic calcification are the elastic tissues of heart, blood vessels, skin, and eyes.

Pseudoxanthoma elasticum (PXE) is an autosomal recessive disease, which was described in 1881 by a French dermatologist. In 2000, it was first recognized that mutation in *ABCC6* is responsible for PXE [1]. It is affecting approximately 1:50,000 people worldwide, with the prominent characteristic feature of ectopic mineralization of soft tissues like skin, eyes, and arteries (Figure 1), for which no effective curative treatment is available [2,3]. Moreover, PXE shows similar phenotypic characteristics with other common health problems like kidney diseases (chronic kidney disease (CKD) and nephrocalcinosis) and cardiovascular diseases (coronary heart disease, cardiomyopathy, and dyslipidemia), which makes PXE a complex disorder [4,5].

Different hypotheses were proposed for the factors pathologically involved with PXE. The “Metabolic Hypothesis” stated that decrease or loss of ABCC6 functionality especially in the liver may lead to a decrease in some circulating factors in the blood stream, which should be responsible for preventing ectopic mineralization of soft tissues. The “PXE Cell Hypothesis” stated that absence of ABCC6 in PXE tissues leads to an alteration in cell proliferation due to changes in the biosynthetic pathway and alters cells to extracellular matrix interactions. The most recent “ATP Release Hypothesis” stated that ABCC6 mediates the efflux of ATP in extracellular milieu, where it is hydrolyzed into AMP and pyrophosphate and prevents the mineralization of soft tissues [1].

Previous studies of serum analysis either from the *ABCC6* knock-down mouse model or from PXE patients showed an inability to prevent calcium and phosphate deposition and suggested that PXE is a metabolic disease with very slow onset [1,6,7]. It should be noted that the tissues, which mostly express ABCC6, are the liver and, to some lesser extent, the kidney and differ from those in which ectopic mineralization is mostly evident, namely soft tissues of skin, eyes, and the cardiovascular system. The origin of this apparent paradox has not been explained yet (Figure 1) [8].

On the basis of our previous studies of the ABCC6 transporter in hepatic cells, the present review is focused on lightening changes in cellular function associated with ABCC6 transporter activity.

## 2. Structural Properties of ABCC6 Transporter

The ATP-binding cassette (ABC) transporters are a well-known family and ubiquitously found in all living organisms. About 50 different types of ABC transporters have been recognized in humans, divided into seven subfamilies (ABCA to ABCG) based on their structure and genetic sequences. Among them we can find both half transporters, with only one transmembrane domain (TMD) and nucleotide-binding domain (NBD), and full transporters, with two TMDs and NBDs [9,10]. ABCC6 belongs to the ATP-binding cassette (ABC) transporter subfamily C and has been found to be highly expressed in basolateral plasma membrane of hepatocytes and, to some lesser extent, in the proximal tubules of the kidney [11].

The *ABCC6* gene has 31 exons and encodes a protein of 1503 amino acid residues, MRP6. It is made-up of two nucleotide-binding domains (NBD1 and NBD2) and two transmembrane domains (TMD1 and TMD2) with an additional auxiliary NH2-terminal transmembrane domain known as TMD0, which is connected with the canonical components through the cytoplasmic L0 loop [12,13]. The NBDs of ABC transporters consist of different conserved motifs which bind and hydrolyze ATP. Like in other transporters, ABCC6 NBDs domains have Walker A motif (P loop), Walker B motif (Mg^2+^ binding site), histidine loop (Switch region), signature motif (C loop), Q loop (between Walker A and C-loop), and D loop (involved in the intermolecular interactions at the NBD dimmer interface). In the general process of dimerization, the Walker A/B motifs of one NBD are aligned with the C-loop of the other in a yin-yang fashion, forming a sandwich with two molecules of ATP in the middle, which are hydrolyzed in the catalytic cycle. Dimerization induces conformational changes in the TMDs, which are reversed by hydrolysis of ATP, leading to efflux of molecules [1,14,15]. In this situation, the ATP should be sandwiched between two sequence/structural motifs that are both rigorously conserved and form a stable homodimer [16]; this is not always possible with the human transporters, because one of the two NBDs is degenerate and the sequence of motifs which are responsible for hydrolysis of ATP is not identical or canonical [14].

**Figure 2:**
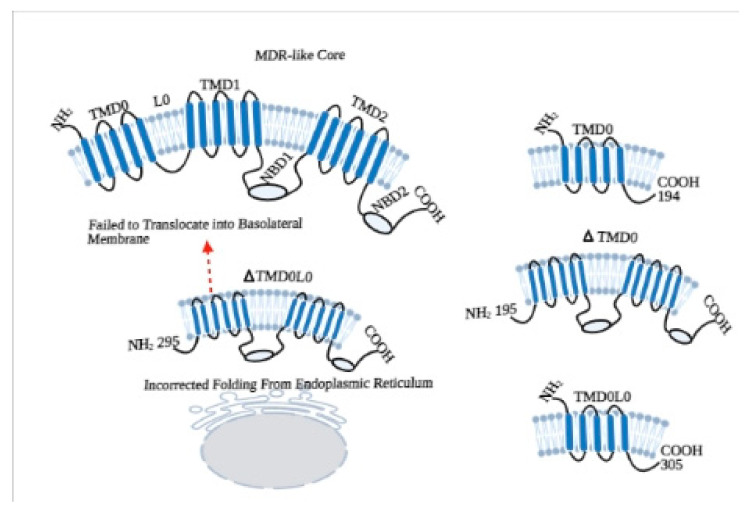
Topology of ABCC6 transporter [20].

In this context to elucidate the role of NBDs, different experiments were conducted on NBD1 of ABCC1/MRP1, which not only has a high level of homology with ABCC6/MRP6 but also shows a superposition of substrates [17]. Studies revealed that the NBDs of MRP1 are not functionally identical, as both the NBDs bind ATP but only NBD2 is involved in hydrolysis. Moreover, the ATP binding affinity of NBD1 of MRP1 is not completely dependent on NBD2 but the binding of ATP to NBD2 is highly dependent on NBD1 [18,19]. In respect of this, we have gone through by a series of experiments to identify the functional roles of NBDs, TMD0, and L0 loop of ABCC6 (Figure 2).

In order to evaluate the functioning of NBDs, we have first characterized the NBD1 of ABCC6/MRP6. In this experiment, by using the E748-A785 fragment of MRP6-NBD1, we have compared the helical structure and ATP binding properties of wild type and the R765Q mutated sequence, which is present in PXE patients. The study with circular dichroism analysis revealed that, in both the wild type and mutated (R765Q) NBD1, E748-A785 peptides adopted α-helical conformations, and the helical content was almost the same for both the peptides in aqueous solutions of trifluoroethanol (TFE). No differences in the length of helices have been found between the peptides in NMR spectroscopy. Moreover, Fluorescence Spectroscopy showed no significant difference in ATP binding capacity of both the peptides. These findings suggest that occurrence of PXE symptoms in R765Q mutated patients might be due to different kind of interactions [21].

In subsequent investigations, we have undergone two different experiments with NBD1 and NBD2, because mutational studies of PXE patients showed that domain-domain interaction is important for proper working of ABCC6 transporter and there is a functional difference between both NBDs of MRP6 [22]. In the first study, we constructed the full-length NBD1 (residues from Asp-627 to Leu-851) and short-length NBD1 (residues from Arg-648 to Thr-805) without some key residues; then differences in helical structure, ATP binding, and hydrolysis of both polypeptides were analyzed. Interestingly, both the polypeptides assumed predominantly α-helical conformation. However, only long-length NBD1 showed β-strand conformation, while short-length NBD1 showed higher helix content, which suggested that the sequences D627-H647 and T806-L851, which are only present in the long-length polypeptide, assume a β-strand conformation, similarly to the corresponding regions of MRP1. Although fluorescence quenching experiments revealed that both the polypeptides have the affinity to bind nucleotides (ATP and ADP), the long-length NBD1 showed a higher affinity to bind ATP than the short-length NBD. In addition, no hydrolytic activity was found in short-length NBD1 compared to long-length NBD1 during spectrophotometric analysis of ATPase activity. These findings suggest that short-length NBD1 lacks of some essential residues, which are responsible for ATP hydrolysis. Whereas, long-length NBD1 form homodimer in the presence of ATP, which is the property of half-transporters and physiologically, it is not possible with the full-transporter where NBD1 interacts with NBD2 [12].

In order to explore how the ABCC6 transporter works in-vivo, we have constructed long-length (Thr-1252 to Val-1503) and short-length NBD2 (Val-1295 to Arg-1468), and we created homology models for homodimer of NBD1 and NBD2, and heterodimer of (NBD1-NBD2) [23]. Circular dichroism spectra of long-length NBD2 revealed that it is more structured and contains α-helical conformation. The nucleotide (ATP and ADP) binding affinity of NBD1 and NBD2 were found to be the same during fluorescence quenching analysis. The ATPase activity of both homo and heterodimer were different, as the amount of inorganic phosphate (Pi) produced by NBD2 was lesser then NBD1. Moreover, addition of NBD2 reduced the NBD1 hydrolytic activity. These findings suggest that NBD2 is well structured (it contains α-helix and β-strands) and binds the nucleotides efficiently, but the ATPase activity of NBD2 is lower compared to the NBD1; this reflects that NBD2 is not able to form a functionally active homodimer, as supported by in-silico structural analysis. In addition, decreased ATPase activity of combined NBD1-NBD2 compared to NBD1 alone suggests that NBD1 and NBD2 work together to form a stable heterodimer and function in a regulated manner, whereas NBD1 alone works in an uncontrolled manner. This can be justified by the in-vivo functioning of the transporter [24], where ATP is hydrolyzed after the binding of substrate on TMDs and leads to conformational changes in membrane domains that activate the NBDs.

## 3. Roles of Additional TMD0 and L0 Domains

**Figure 3:**
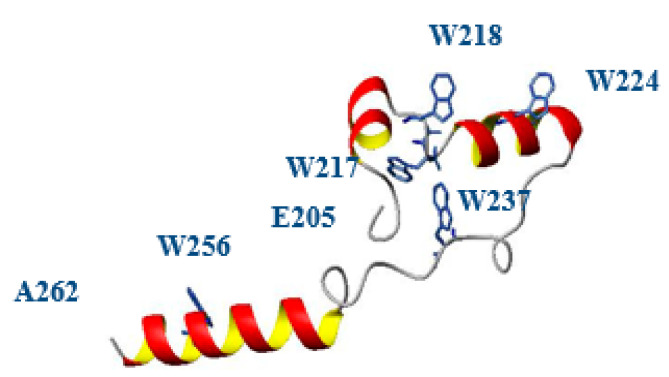
Homology model of L0 loop. The loop includes the region between residues E205 and A262. The PDB structure 5UJ9 corresponding to MRP1 was used as template.

The functional role of TMD0 in other proteins like MRP1, MRP2, SUR1 is well defined, which may be involved in stabilization and retention of the transporter in the plasma membrane or in regulation of channel activities. By topological modeling of TMD0 of MRP6, we demonstrated that it contains five transmembrane domains with the N- and C-termini on the external and cytoplasmic side, respectively. These TMs are inserted into the membrane individually on the basis of hydrophobicity and without affecting each other. In addition, we have also found that disease-causing mutations did not affect the membrane insertion of these TMs [25]. In a further study, we have done the structural and functional characterization of L0 loop of ABCC6. We have found that L0 loop of ABCC6 is well structured (Figure 3) as it contains aromatic residues, and three α-helical regions; it resembles the homologous L0 loops of other MRPs and is responsible for plasma membrane localization of TMD0 [13]. However, to understand the exact role of TMD0 and L0 loop (N-terminal Region) of ABCC6, we have constructed two variants of N-terminal lacking TMD0 (ΔTMD0) and a variant lacking both TMD0 and L0 (ΔTMD0L0). Interestingly, we have found that ΔTMD0L0 not only failed to exhibit transport activity, but it was also not able to localize at the basolateral side of the plasma membrane, which reflects that L0 loop is important for both activities. Thus, these findings suggest that L0 not only contains a basolateral sorting signal but that L0 also contributes to folding ABCC6 into a cellular sorting-competent state, which is necessary to pass the endoplasmic reticulum (ER) quality control system and continue through the secretory pathway. In addition, we also found that L0 loop of ABCC6 interacts with the ion channels like other members of ABCC family and might be involved in the modulation of Ca^2+^ channels of plasma membrane [20].

## 4. The Decrease or Loss of ABCC6 Functionality Changes Extracellular Environment

In early pathological investigations of PXE, Iliás et al. in 2002 proposed that ABCC6 is a transporter of organic anions and actively transports glutathione conjugates, including leukotriene C4 and N-ethylmaleimide S-glutathione (NEM-GS), and their abolishment due to missense mutation in ABCC6 gene is responsible for PXE [26,27]. In the same line of thinking, Borst and co-workers in 2008 proposed that ABCC6 transporter mediates the efflux of vitamin-K as a glutathione or glucuronide conjugate and is involved to prevent calcification of soft tissues [28]. However, in the further investigations, it was found that ABCC6 is not a transporter of vitamin-K, as its administration in PXE mouse model (ABCC6−/−) was not able to prevent the mineralization [29,30]. As discussed above, Jansen et al. in 2013 proposed that ABCC6 transporter mediates the efflux of ATP and indirectly produces PPi to prevent ectopic mineralization [2]. However, we have demonstrated that PXE is a complex metabolic disease with the reprogramming of crucial genetic factors in the absence of ABCC6 transporter activity [31,32,33].

**Figure 4:**
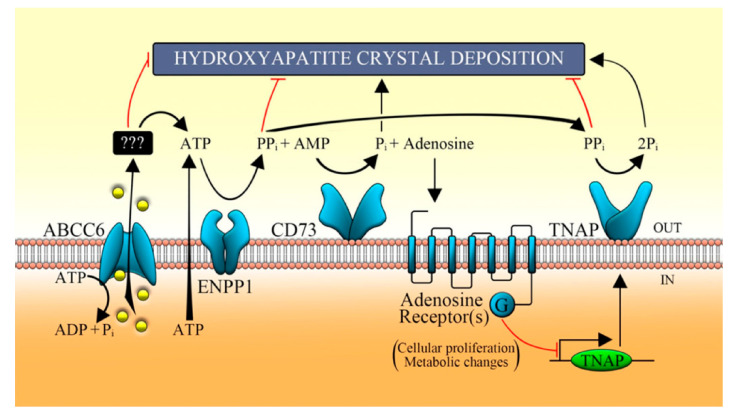
Proposed pathomechanism of PXE. ABCC6 transporter mediates the efflux of ATP, which is metabolized by some ecto-nucleotidases (such as ENPP1) in AMP, which in turn is converted in adenosine and Pi by CD73. In the extracellular milieu, nucleotides regulate the activity of TNAP through the purinergic pathway and prevent the ectopic mineralization [32]. ABCC6, ATP-binding cassette, sub-family C, member 6; ENPP1, ecto-nucleotide pyrophosphatase/phosphodiesterase type I; CD73, cluster of differentiation 73; TNAP, tissue non-specific alkaline phosphatase; Pi, inorganic phosphate; PPi, inorganic pyrophosphate [35].

In order to better understand the pathomechanism of PXE (Figure 4), we stably knocked down the *ABCC6* gene in HepG2 cells by using shRNA, and its associated transcriptional/genetic changes were studied. We first examined the production of reactive oxygen species (ROS), which are supposed to increase according to the previous PXE fibroblast studies [34]. On the contrary, in the *ABCC6* knockdown HepG2 cells, the ratio of GSH/GSSG has been found to be increased whereas a significant decrease in ROS level was observed, which means that knockdown cells resembled the reductive stress, which is also required by proliferating cells.

**Figure 5:**
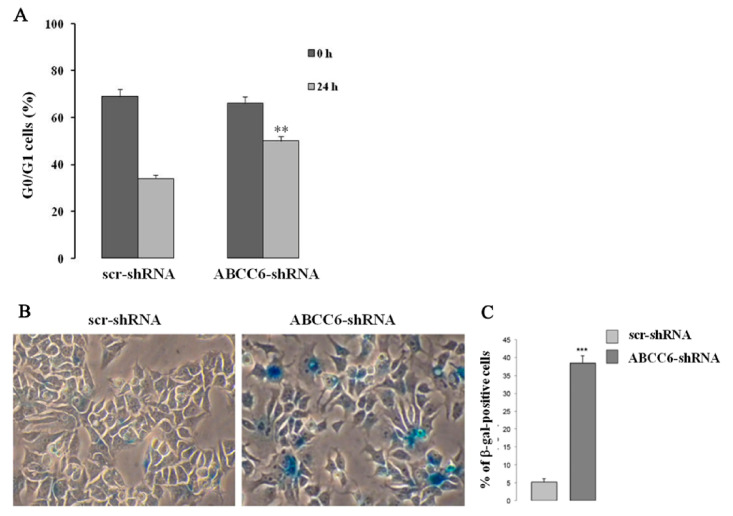
Pictorial presentation of senescent-like phenotype in ABCC6 knockdown HepG2 cell. (**A**)—For the cell cycle analysis, the cells were synchronized at the G1 phase by serum deprivation for 24 h, restimulated with serum for 24 h, and analyzed using flow cytometry after BrDU and PI staining. The percentage of control (scr-shRNA) and ABCC6 knockdown cells (ABCC6-shRNA) in G0/G1 was recorded. (**B**)—Representative images (40 × magnification) of senescence-associated β-galactosidase staining in control and ABCC6 knockdown cells. (**C**)—Quantitative analysis of positive β-galactosidase-stained cells. Data were generated from three independent experiments performed in triplicate and are shown as means ± SD. Statistical analysis was performed using unpaired Student’s *t* test: ** *p* < 0.01 and *** *p* < 0.001 [33].

However, we found significant delay in G1 to S transition and slower cell growth in ABCC6 knockdown HepG2 cells (Figure 5). In addition, expression of cyclin-dependent kinase inhibitor (CDKI) p21, which negatively regulates the activity of CDK and is required for the cell entry into the different cycle phases, was found increased in knockdown cells. Moreover, the expression of lamin A/C, which is required to maintain the strength of the nucleus and is pathologically involved in aging process, was decreased in those cells [33].

Interestingly, knockdown HepG2 cells were shown to have a decreased expression of the ecto-5′-nucleotidase (*NT5E* or CD73), which regulates the conversion of AMP to adenosine and Pi (inorganic phosphate). Additionally, nonspecific alkaline phosphatase (TNAP), whose activity normally maintains Pi/iPP ratio accelerating the mineralization, was found to be increased in knockdown HepG2 cells. These results suggest that the absence of transporter activity in the hepatic cells decreases the *NT5E* expression and increases the pro-mineralizing TNAP activity, which is also clinically found in patients with arterial calcifications due to deficiency in CD73 (ACDC) [31]. It is clear from the knockdown study that the absence of transporter activity leads to alteration in gene expression, which is required to provide PPi to prevent mineralization. However, in a further study, we also found that ABCC6 plays a crucial role to activate the purinergic pathway, which is important to maintain proper cellular function. In this study, pharmacological inhibition of ABCC6 by probenecid down-regulated the expression of CD73. On the contrary, the expression of CD73 was found increased after the application of adenosine and ATP, which strengthens the idea that ABCC6 not only mediates the efflux of ATP but also regulates the purinergic system [35].

## 5. Intracellular Consequences Associated with ABCC6 Transporter Activity in HepG2 Cells

It is widely evident that changes in nuclear lamin expression are associated with cellular senescence and age-related diseases [36]. However, cells undergoing aging also adapt a phenomenon to proliferate inappropriately, migrate, and colonize, which are the hallmarks of cancer cells [37]. In both cases, changes in lamin expression traduce in morphological changes in nuclei, which are known as “nuclear atypia”. Contrarily, induction of cellular senescence is recognized as an important tumor-suppressive mechanism [38].

**Figure 6:**
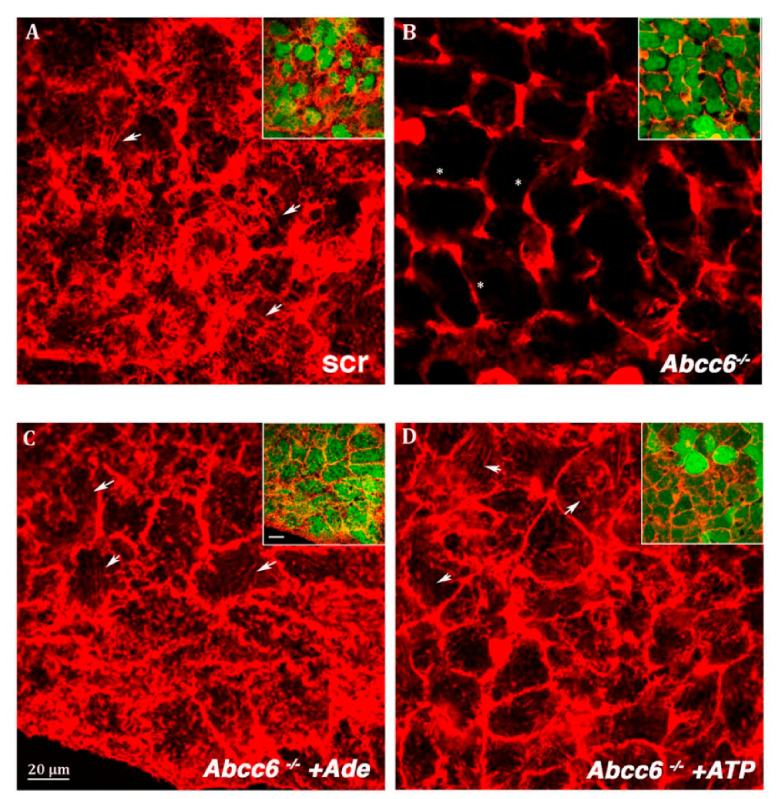
Representative confocal image of (**A**)—scrambled HepG2 cells; (**B**)—Abcc6-shRNA.HepG2 cells; (**C**)—Abcc6-shRNA HepG2 cells treated with 500 µM ATP; (**D**)—Abcc6-shRNA HepG2 cells treated with 100 µM adenosine. F-actin was stained with Texas Red-phalloidin. In the insets superposition of cytoskeleton (red) and EGFP (green) to monitor the infection efficiency. The scale bar in the inserts is 40 µm [32].

**Figure 7:**
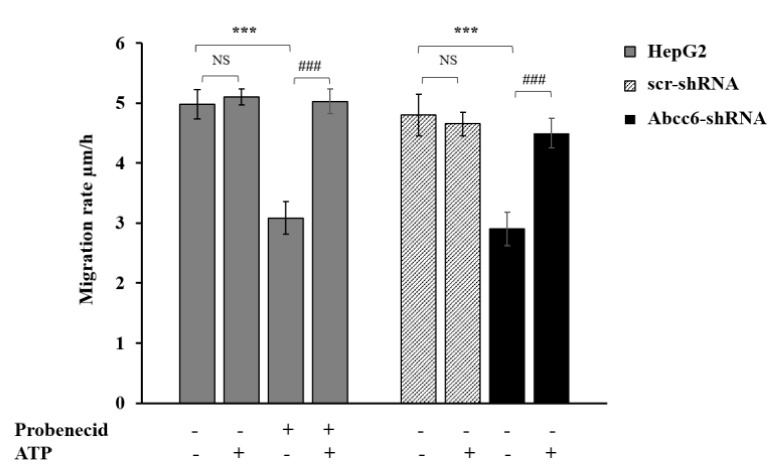
Effect of probenecid and ABCC6 silencing on the migration rate of HepG2 cells. Cells were treated with 250 μM probenecid for 48 h (gray plain bars, Probenecid+). DMSO-treated cells were used as the control (gray plain bars, Probenecid-). A total of 500 µM ATP was added to either the control cells (gray plain bars, Probenecid-, ATP+) or to probenecid-treated cells (gray plain bars, Probenecid+, ATP+). HepG2 cells were transduced with scrambled shRNA (grey-texturized bars, scr-shRNA) or with specific ABCC6-shRNA (black bars, ABCC6-shRNA). A sample of 500 µM ATP was added to either control cells (grey-texturized bars, scr-shRNA, ATP+) or to ABCC6 silenced cells (black bars, ABCC6-shRNA, ATP+). Data are expressed as mean ± standard error (SE) of three replicates from three independent experiments and were analyzed by one-way ANOVA followed by Dunnett’s post hoc: *** *p* < 0.001 probenecid-treated cells vs. control cells in the absence of ATP and ABCC6-shRNA vs. scr-shRNA in the absence of ATP; ### *p* < 0.001 probenecid + ATP-treated cells vs. probenecid-treated cells and ABCC6-shRNA cells + ATP vs. ABCC6-shRNA cells without ATP. NS, not significant [32].

In previous studies with *ABCC6* knockdown HepG2 cells, we have found decreased cell growth and lamin A/C expression [33]. Interestingly, in the recent study, pharmacological inhibition of ABCC6 by probenecid or its knockdown in HepG2 cells not only decreased the amount of extracellular ATP content but also decreased the expression of CD73 and lamin A/C proteins. Lamins are the most important structural component of the cytoskeleton and are required to maintain cells in a proper shape. In this context, we examined whether down-regulation of lamin expression affected the actin filaments, required for cytoskeleton rearrangement and cellular movement. In this study, a typical organization of actin filament in filopodia, which is a hallmark of moving cells, was absent in both probenecid-treated and *ABCC6* knockdown HepG2 cells (Figure 6). However, administration of adenosine or ATP restored the normal architecture of filopodia and migration rate (Figure 7). This finding indicates that ABCC6 can be a potential therapeutic target for anti-metastatic treatment and, with coordination of the purinergic system, also regulates the intracellular functions [32].

## 6. Involvement of ABCC6/MRP6 in Drug Resistance

It is well known that ABC transporters play an important role to maintain cellular physiology by transporting different substrates. On the other hand, despite the pathological involvement in certain crucial diseases, these transporters are also involved in drug resistance [39]. Similarly, ABCC family consisted of 13 members, which are involved in the transportation of different substrates. Nine of them are also implicated in the resistance to a variety of chemotherapeutic agents. Martin G. et al., in a study on Chinese hamster ovarian cancer cells (CHO), demonstrated that ABCC6 is not only able to mediate the efflux of glutathione conjugate but is also involved in resistance to some natural anti-cancer drugs, like doxorubicin, etoposide, actinomycin D, daunorubicin, and cisplatin [27]. Tyrosine Kinase Inhibitors (TKIs), like nilotinib and dasatinib, are effective treatments available for Chronic Myeloid Leukaemia (CML) and found to interact with ABCB1 and ABCG2. However, a recent study suggested that nilotinib and dasatinib might be substrates for ABCC6, whose over-expression is responsible for resistance of both TKIs [40].

To investigate the possible involvement of different transporters in drug resistance, we investigated the mRNA expression of ABCC6 and other ABC transporters mainly involved in drug resistance, such as ABCB1, ABCC1, and ABCG2, in the bone marrow samples obtained from acute myeloid leukemia (AML) patients. The samples obtained after diagnosis revealed no difference in the expression level of ABCC6 and ABCB1 between healthy control and AML patients. The expression of ABCC1 from AML patients was higher compared to the healthy individual. Moreover, the expression of ABCG2 was always found down-regulated in AML patients compared to controls. We also observed the age- and gender-associated changes in the expression level of the genes. Interestingly, ABCB1, ABCC1, and ABCC6 were less expressed in older patients compared to younger ones, whereas ABCC6 and ABCC1 were highly expressed in female patients. Additionally, only ABCG2 expression was found higher after chemotherapy, while no variation was found in ABCC1, ABCB1, and ABCC6. However, the expression of ABCC6 and ABCB1 was found to be up-regulated after the treatment with Trichostatin A (an inhibitor of histone deacetylase) and 5-Aza-2′deoxycytidine (an inhibitor of DNA methyltransferase) in AML cell line HL-60. These data reveal that changes in expression of ABCG2 before and after the treatment can be related to disease or as a therapeutic marker. On the other hand, the expression of ABCC6 and ABCB1 is transcriptionally or epigenetically controlled [41].

In addition, a study conducted by Jeon H-Metal [42] suggested that an inhibitor of differentiation 4 (ID4) increases the SOX2-mediated expression of ABCC6 and ABCC3 in glioma stem cells (GSC), which have the potential to initiate a brain tumor, show resistance to chemotherapy, and are responsible for higher recurrence rates of Glioblastoma multiforme (GBM) [43].

Drug resistance is the biggest problem for cancer therapy, for which many molecules have been synthesized and tested, but MDR in cancer therapy still persists. In order to identify the promising molecules to inhibit MRP6 and mitigate marginal drug resistance, we tested 8-(4-chlorophenyl)-5-methyl-8-[(2Z)-pent-2-en-1-yloxy]-8H-[1,2,4] oxadiazolo [3,4-c][1,4] thiazin-3, also known as 2C and structurally similar to diltiazem. Surprisingly, the efflux of doxorubicin was reduced in 2C-treated cells. Moreover, cell esterase activity and H3 histone acetylation were reduced in 2C-treated cells, which suggests that 2C is not only able to mitigate drug resistance but also able to inhibit nucleophilic substitution reactions [44].

These findings confirm that ABCC6 is not only involved in the progression of the genetic disease PXE but is also involved in resistance to many anti-cancer agents [45,46].

## 7. Conclusions

Different hypotheses were given to elucidate the physiological substrates for the ABCC6 transporter and its pathological involvement in PXE. However, mechanistic details for *ABCC6* mutations leading to ectopic mineralization were yet to be resolved [6,47,48]. In our studies, we not only investigated the structural components of ABCC6 transporter but also lighten the mechanisms, which might be involved in ectopic mineralization. In our experiments, knockdown of *ABCC6* or its inhibition by probenecid decreased the extracellular ATP concentration and altered the expression of *NT5E* and *TNAP*. By these findings, we can propose that the lack of ABCC6 transport activity could lead to a pro-mineralizing state through alteration of extracellular purine metabolites, which ultimately affect TNAP enzymatic activity.

Moreover, in both knockdown and probenecid-treated HepG2 cells, the expression of lamin was found decreased, which suggests that reduced ABCC6 transport activity also leads to cell senescence in PXE and can be beneficial to prevent cancer progression. Interestingly, in both the knockdown and probenecid-treated HepG2 cells, we found a decrease in migration rate, which is restored after ATP administration.

Collectively, these findings suggest that the ABCC6 transporter is not only required to maintain some important circulatory factors like PPi, but is also required to maintain proper functioning of other factors, which are involved in the conversion of extracellular nucleotides, and to feed purine pool to maintain homeostasis between Pi and PPi ratio. Moreover, our studies strengthen the idea that the ABCC6 transporter is not only involved in PXE pathophysiology, but can also be considered a target for anti-cancer therapy.
